# Effects of Testosterone Hormone on the Sexual Aspect of Postmenopausal Women: A Systematic Review

**DOI:** 10.7759/cureus.68046

**Published:** 2024-08-28

**Authors:** Julio G Rojas-Zambrano, Augusto R Rojas-Zambrano

**Affiliations:** 1 Gynecology, Dr. Regeneracion, Guayaquil, ECU; 2 General Medicine, Dr. Regeneracion, Guayaquil, ECU

**Keywords:** sexual dysfunction, testosterone deficiency, testosterone insufficiency, testosterone, menopause women

## Abstract

A decrease in testosterone levels in women during the postmenopausal period is associated with a wide range of signs and symptoms that can negatively impact their sexual quality of life. Due to this, it is crucial to understand and address this hormone deficiency with appropriate medication. To summarize the effects of testosterone treatment on the sexual aspects of postmenopausal women, PubMed was searched from 1974 to the present using the following MESH terms: (((testosterone) OR (androgens)) OR (testosterone deficiency)) AND (sexual dysfunction) AND (postmenopausal women) AND (current therapy replacement))). The inclusion criteria were studies with observational and experimental approaches that evaluated the mechanism of action of testosterone in postmenopausal women. The updated data indicate that testosterone therapy alleviates many of the signs and symptoms related to sexual dysfunction in menopausal women. However, the evidence is limited due to the small sample sizes and the relatively few studies on this topic. As our understanding of the relationship between testosterone and postmenopausal women advances, there has been significant development in the use of this hormone. To validate these findings and ensure they are generalizable, further randomized controlled trials are essential. Future studies should focus on confirming the efficacy of testosterone therapy.

## Introduction and background

Based on the North American Menopause Society, menopause is considered normal when it is marked by the final menstrual period, diagnosed after 12 consecutive months of natural amenorrhea with no identifiable medical reason [1]. Women typically experience a range of signs and symptoms during menopause, with the most common being hot flashes, night sweats, sleep disturbances, vaginal atrophy, and dyspareunia [2-3]. In postmenopausal women, most experience vulvovaginal atrophy. Due to this condition, they may develop sexual dysfunction, including loss of desire, decreased lubrication, and loss of orgasm, leading to psychophysiological changes and interpersonal issues [4].

Testosterone is a crucial hormone that plays a significant role in maintaining the function of the female genital apparatus, including vaginal lubrication, which is essential for sexual health [5]. Research has shown that testosterone levels in women are typically higher than those of estradiol [6]. Testosterone is produced by key organs such as the ovaries and adrenal glands. Once released into the bloodstream, it binds to sex hormone-binding globulin, which has a higher affinity for testosterone than albumin [7]. When testosterone binds to androgen receptors, it triggers the activation of genes responsible for various functions, including sexual function [8].

During menopause, levels of androgens, including testosterone, decline due to the aging effects on adrenal and ovarian functions. This reduction can lead to decreased sexual motivation [9]. Given this background, it is hypothetically believed that a decrease in testosterone could be a reversible cause of hypoactive sexual desire disorder (HSDD). Currently, there are guidelines for the management and use of testosterone replacement therapy (TRT) in women during the menopausal period [10].

In the United States, menopausal and postmenopausal women frequently experience female sexual dysfunction (FSD) and HSDD, which are among the most commonly reported conditions in studies [11]. It is crucial to recognize that sexual dysfunction not only affects the women themselves but also has a significant impact on their relationships [11].

This systematic review aims to summarize the evidence regarding the effects of testosterone on the sexual health of postmenopausal women. It is important to recognize that hormone levels, including testosterone, fluctuate throughout different stages of life. Understanding these variations is crucial for comprehending the impact of testosterone on sexual health during postmenopause.

## Review

We conducted a literature search following the guidelines of the Preferred Reporting Items for Systematic Reviews and Meta-Analyses (PRISMA) [12]. It is important to note that this research does not require ethical approval, as it involves reviewing existing published articles on patient data. A comprehensive search was performed in the PubMed database using the following MeSH terms: (((testosterone hormone) OR (androgens)) OR (testosterone deficiency)) AND (sexual dysfunction) AND (postmenopausal women) AND (current therapy replacement)).

**Figure 1:**
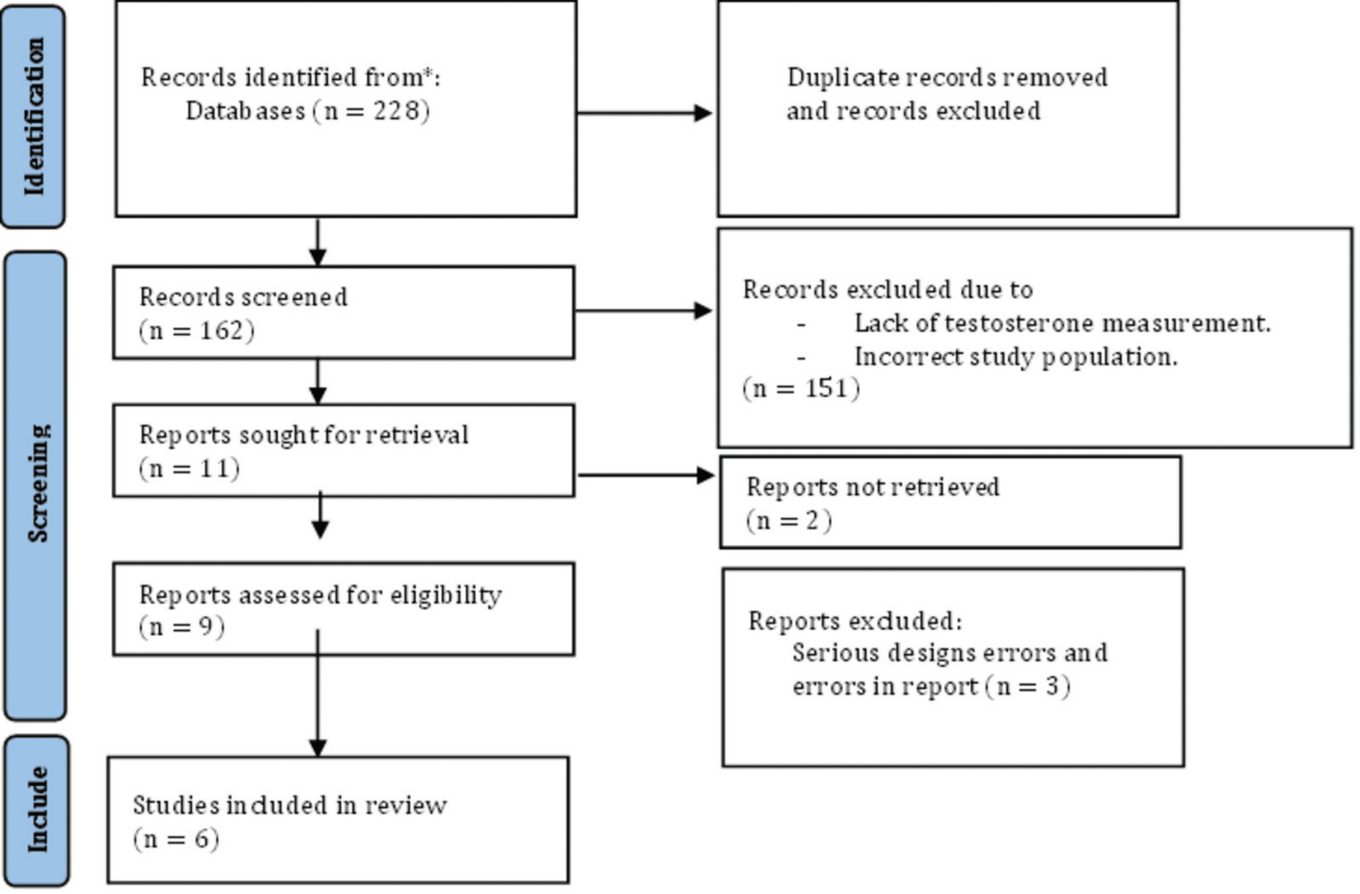
PRISMA flow PRISMA: Preferred Reporting Items for Systematic Reviews and Meta-Analyses

We used data from 1974 to the present. Publications with observational and experimental designs on humans that evaluated the effect of testosterone on sexual aspects were included as part of the inclusion criteria. Studies involving another hormone (estrogen) or focusing on women under the age of 40 were excluded. Figure 1 shows the selection process [12]. To provide a better understanding of the data, we used a table to summarize all the information collected, comparing different aspects of sexual health.

Results

Selection and Characteristics

"Our study began with an initial set of 228 articles, as illustrated in Table 1. After reviewing the titles and abstracts, 66 articles were removed due to duplication. Following a full-text review, an additional 156 records were excluded. In the end, six articles remained and were subjected to further examination.

**Table 1:** List of articles HSDD: hypoactive sexual desire disorder, TTh: testosterone therapy, FSD: female sexual dysfunction, TRT: testosterone replacement therapy

Author(s), years	Study design	Aim of study	Setting and participants	Results and findings
Sarmento et al. (2022) [13]	Systematic review	To evaluate the efficacy of the hormonal and nonhormonal approaches to symptoms of sexual dysfunction and vaginal atrophy in postmenopausal women	55 articles	This review suggests that vaginal hormone therapy with DHEA could positively influence sexual desire and sexual function
Martimbianco et al. (2020) [14]	Systematic review	To assess the effectiveness and safety of *Tribulus terrestris* in treating FSD	5 randomized controlled trials	Increase in sexual function scores
Jayasena et al. (2019) [15]	Systematic review	Investigating the efficacy and safety of testosterone therapy for FSD in postmenopausal women	69 articles	In summary, although the majority of studies suggest that testosterone helps reduce symptoms of sexual dysfunction irrespective of the method of administration, this conclusion is not universally supported by all research
Achilli et al. (2017) [16]	Systematic reviews and meta-analysis	To systematically review and summarize the existing evidence related to the efficacy and safety of transdermal T in postmenopausal women for the treatment of HSDD	7 randomized controlled trials.	The T group experienced notably higher levels of satisfying sexual episodes, sexual activity, orgasms, and desire, as well as significant improvements in Personal Distress Scale scores, they reported more androgenic side effects, including acne and increased hair growth, compared to the placebo group
Khera (2015) [17]	Narrative review and expert opinion	To provide an overview of the current literature regarding the use of TTh for the treatment of FSD	66 articles	Testosterone therapy has been demonstrated to enhance various aspects of FSD, including sexual desire, arousal, pleasure, and overall satisfaction, although, testosterone therapy can lead to side effects like acne and hirsutism, there is no strong evidence suggesting that it increases the risk of cancers such as breast or endometrial cancer
Uloko et al. (2022) [10]	Narrative review	To highlight the use of TRT in the management of the postmenopausal woman experiencing symptoms of HSDD	71 articles	Conclude that testosterone is a vital hormone in women in maintaining sexual health and function

Data

The systematic review included a total of six studies, all of which had a positive impact on the sexual aspects of postmenopausal women, reducing their sexual dysfunction and improving sexual desire and overall satisfaction [10,13-17]. The improvement was similar to that observed in patients who had undergone surgical menopause, where the administration of testosterone also improved their sexual dysfunction [18].

Discussion and perspective

Current evidence indicates that testosterone therapy for FSD can improve not only dyspareunia and vaginal dryness but also sexual desire and orgasm [19]. However, this treatment is not suitable for all women, particularly those with conditions such as breast cancer, endometrial cancer, or deep venous thrombosis. Furthermore, there is limited evidence regarding the effectiveness of nonhormonal therapies in improving orgasm, lubrication, and overall sexual satisfaction in postmenopausal women [2].

The current review suggests that testosterone therapy may positively affect sexual desire and function across all areas, not just in reducing dyspareunia and vaginal dryness [19]. Nonetheless, some studies indicate that testosterone might not be appropriate for cases of low sexual desire [20]. Therefore, the results of this study should not be broadly applied until additional randomized clinical trials are conducted to validate and reinforce the evidence.

Given that testosterone therapy has shown benefits in sexual health [19], it is crucial to recognize that its use should be approached with caution. More randomized clinical trials are necessary to establish its safety and efficacy, especially considering the potential side effects that have been observed [20].

## Conclusions

This comprehensive review underscores the potential benefits of testosterone therapy in addressing FSD and HSDD in postmenopausal women. Our analysis, grounded in a review of six studies, emphasizes that testosterone therapy can notably improve dyspareunia and vaginal dryness, as well as boost sexual desire and orgasm. This aligns with the growing body of evidence supporting testosterone's role in improving various aspects of sexual health, particularly in those suffering from vulvovaginal atrophy and diminished sexual motivation.

The results of this review suggest that testosterone therapy could have a profoundly positive impact on sexual health across multiple domains, extending beyond the relief of specific symptoms like dyspareunia and vaginal dryness. However, conflicting evidence regarding its efficacy for low sexual desire indicates the need for a nuanced approach to treatment.

To fully validate these findings and ensure they can be generalized, further randomized controlled trials are essential. These future studies should aim to confirm the efficacy of testosterone therapy, evaluate its long-term safety, and clarify its role in the broader context of sexual health management for postmenopausal women.
